# Glutaraldehyde Cross-Linking of Salt-Induced Fibrinogen Hydrogels

**DOI:** 10.1021/acsbiomaterials.4c01412

**Published:** 2024-10-18

**Authors:** Dominik Hense, Oliver I. Strube

**Affiliations:** Institute for Chemical Engineering, University of Innsbruck, Innrain 80-82, Innsbruck, AT 6020, Austria

**Keywords:** fibrinogen, fibers, fibrillogenesis salt-induced, hydrogels, cross-linking, dissolution, glutaraldehyde

## Abstract

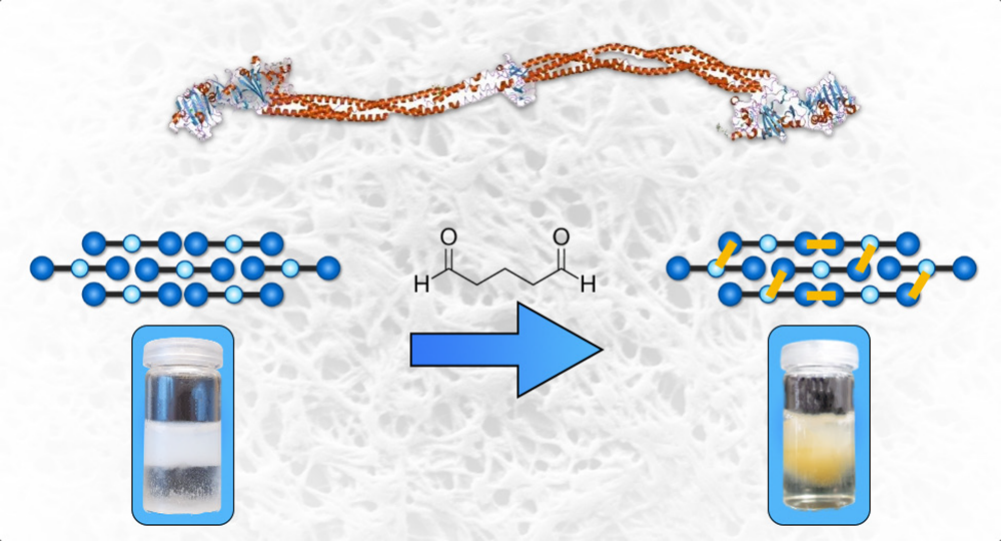

Covalent cross-linking is a common strategy to improve the mechanical properties of biological polymers. The most prominent field of application of such materials is in medicine, for example, in the form of bioprinting, drug delivery, and wound sealants. One biological polymer of particular interest is the blood clotting protein fibrinogen. In the natural process, fibrinogen polymerizes to fibrous hydrogel fibrin. Although the material shows great potential, its costs are very high due to the required enzyme thrombin. Recently, we introduced several approaches to trigger a thrombin-free fibrillogenesis of fibrinogen to a fibrin-like material. Inspired by the natural pathway of blood clotting in which covalent cross-linking stabilizes the clot, this “pseudofibrin” is now developed even further by covalently cross-linking the fibers. In particular, the effect of inexpensive glutaraldehyde on fiber morphology, rheological properties, and irreversible gel dissolution is investigated. Additionally, new insights into the reaction kinetics between fibrinogen and glutaraldehyde are gained. It could be shown that the fibrous structure of pseudofibrin can be retained during cross-linking and that glutaraldehyde significantly improves rheological properties of the hydrogels. Even more important, cross-linking with glutaraldehyde can prevent dissolution of the gels at elevated temperatures.

## 1 Introduction

Biological materials have gained interest in many different areas of our lives. This ranges from “greener” product design to high-performance medical applications. The latter profit from biological polymers such as polysaccharides and proteins, often even in combination. One of these materials is fibrin, the fibrous protein network emerging during blood clotting. Due to its fibrous structure and hydrogel nature, it became a commonly used biopolymer in medical research. Its most famous field of application is fibrin glue, which has been studied for decades due to its great performance and tolerability after surgeries.^1−5^ Other intensively studied areas include drug^6−8^ and cell delivery,^9,10^ cell culturing,^11,12^ and scaffolds for tissue engineering.^13−15^*In vitro* fabrication of fibrin is rather uncomplicated and requires solely the precursor protein fibrinogen and enzyme thrombin. Due to enzymatic cleavage of four oligopeptides, the fibrinogen molecule becomes activated and spontaneously polymerizes to fibers and eventually networks.^16^ The main downsides of fibrin as a material, however, stem exactly from this fabrication route. Especially regarding the costs and the risk of thrombosis, a thrombin-free alternative to fibrin is desirable. Fortunately, the most important properties of fibrin, being its biocompatibility and excellent cell adhesion, stem from the precursor protein fibrinogen itself.^16^

Fibrinogen is a 45 nm long rod-like glycoprotein with a molecular weight of approximately 340,000 Da. It consists of two symmetric half-molecules, which are connected by 29 disulfide bridges in the central region of the molecule.^16,17^ Many approaches have been developed to turn pure fibrinogen into a usable material. Exemplary studies describe nanoparticles for drug delivery,^18^ porous aerogels for bone repair,^19^ and gelation upon addition of specific divalent cations such as Co^2+^ or Fe^2+^.^20^ Moreover, many advances were made in the field of (nongelled) fibrinogen fibers. Although some of them are fabricated via external forces such as electrospinning,^21^ a surprisingly high number of self-assembly processes yield fibrinogen fibers. These can be, for example, dialysis against buffers of low ionic strength,^22^ addition of ethanol under defined conditions,^23,24^ and surface-induced effects.^25−27^

To yield fibrous hydrogels that are actually comparable to fibrin, we previously proposed a novel salt-induced self-assembly process. It can be triggered by kosmotropic anions (e.g., sodium phosphate buffer) under narrow reaction conditions such as a temperature of 5 °C and otherwise salt-free environment.^28^ Exchanging the cation in this process has a high impact on the outcome, so the process could be improved by using MgSO_4_ (to combine kosmotropic anions with divalent cations)^29^ and especially by using Ca^2+^ salts.^30^ Due to their similarity to fibrin, these materials were named “pseudofibrin”.

Especially the approach via kosmotropic anions yields a comparatively soft material, making it hardly suitable for many typical use cases. This issue is also known for fibrin, although to a lower extent. To overcome this limitation here, a common approach is to create composite materials of fibrin, for example, with poly(ethylene glycol),^31^ gelatin,^32^ or hyaluronic acid.^33^ An alternative solution is inspired by the natural blood clotting process. Its last step is the introduction of covalent bonds by the transglutaminase factor XIII, leading to a mechanically and chemically more resistant material.^16^ Although there are some approaches that use exactly this enzyme for stabilization of fibrin materials,^34^ a more common strategy is to use synthetic cross-linkers. The main reason for this is the high cost of transglutaminase, which is even higher than that for thrombin. Common examples of successfully used cross-linkers are formaldehyde,^35^ the biobased genipin,^36,37^ 1-ethyl-3-(3-(dimethylamino)propyl) carbodiimide (EDC),^18^ dicyclohexylcarbodiimide (DCC),^38,39^ and tetranitromethane.^40^ Another frequently used cross-linker is glutaraldehyde (1,5-pentanedial), which reacts with primary amines.^41^ Regarding the mechanism of cross-linking, glutaraldehyde exhibits a remarkably versatile behavior in aqueous solution, which includes, for example, intramolecular cyclization and oligomerization.^42^ Thus, a prediction of its form and exact reaction mechanism is difficult. Besides this, glutaraldehyde cross-linking resembles a fast and uncomplicated way of stabilizing biological materials.^40,43,44^

The present study aims to stabilize the previously introduced pseudofibrin hydrogels with glutaraldehyde to provide a material with properties even closer to natural fibrin. The focus of this work is the anion-induced pseudofibrin gels, which profit the most from such stabilization. After investigating the optimal reaction conditions, the impact of cross-linking will be quantified via rheologic measurements, the kinetics of cross-linking shall be monitored, and a suitable protocol to deactivate the harmful excess glutaraldehyde shall be developed. This work shall give deeper insights into the fibrinogen–glutaraldehyde cross-linking reaction and provide a stabilized material suitable for manifold applications.

## 2 Experimental Section

### 2.1 Materials

Bovine fibrinogen, lyophilized from citrate buffer (20 mmol/L), with a purity ≥95% (of which ≥95% is clottable) was obtained from Merck and stored at 5 °C. Glutaraldehyde was delivered from Merck in the form of a 50 wt % aqueous solution. Lysine hydrochloride with a purity >99% was obtained from Alfa Aesar.

All experiments were carried out in pure water (HPLC grade, specific conductivity max 1 μS/cm) by VWR Chemicals. All other chemicals were of high purity and were purchased from the usual suppliers. Chemicals, especially fibrinogen, were used without any purification steps.

### 2.2 Preparation of Pseudofibrin Hydrogels

A 20 mg portion of fibrinogen was suspended in 4 mL of 5 °C cold pure water (final concentration: 5 g/L). Then, 15 μL of 0.1 mol/L NaOH was added to reach a pH of 7.0. The solution was stirred for 10 min at 300 rpm. Fibrinogen dissolved at this time. The stirring bar was then removed. To trigger fiber formation and therefore gelation, 120 μL of a 500 mmol/L sodium phosphate buffer was added fast but without shaking or stirring. The reaction mixture was carefully placed in the refrigerator (5 °C) for 4 h.

### 2.3 Identification of Optimal Cross-Linking Conditions

Six pseudofibrin hydrogels were prepared as described in 2.2 using a pH 6.5 sodium phosphate buffer. After the gels formed, the fibers were covalently cross-linked. First, the influence of different glutaraldehyde concentrations was studied. For this purpose, three gels were cross-linked by dropwise addition of 120, 210, or 301 μL of a 500 mmol/L glutaraldehyde solution. This results in concentrations of 15, 25, and 35 mmol/L glutaraldehyde, respectively. Cross-linking lasted for 15 min at 5 °C. Afterward, a SEM sample of each experiment was prepared as described in Section 2.10.

Subsequently, the influence of the reaction time was studied. The remaining three gels were cross-linked by the dropwise addition of 120 μL of the 500 mmol/L glutaraldehyde solution. Cross-linking lasted for 30 min, 60 min, or 20 h at 5 °C. After the respective reaction time, a SEM sample was prepared as described in Section 2.10.

An additional experiment was performed to study the influence of the reaction temperature. A pseudofibrin hydrogel was prepared as described above but cross-linked for 15 min at 25 °C. A SEM sample was prepared as described in Section 2.10.

### 2.4 Mechanical Properties of Pseudofibrin Gels before and after Cross-Linking

Two pseudofibrin hydrogels were prepared as described in Section 2.2. After their preparation, they were cross-linked by the dropwise addition of 120 μL of a 500 mmol/L glutaraldehyde solution. Cross-linking lasted for 10 min at 5 °C.

Rheological properties were measured using an Anton Paar MCR 302 in a cone/plate setup at 5 °C. The cone had a diameter of 50 mm, and the distance from the cone to the plate was 0.1 mm. With the first gel, an amplitude scan was performed from 0.01 to 100% shear deformation at 1 Hz. The second gel was used to perform a frequency test in the range from 0.01 to 10 Hz.

Rheological measurements of untreated pseudofibrin and noncovalently cross-linked fibrin are already described in a previous study.^28^

### 2.5 Estimation of Cross-Linking Kinetics and Degree of Conversion

The kinetics and degree of conversion of glutaraldehyde cross-linking were measured by means of UV/vis spectroscopy. A Mettler Toledo UV 5 instrument was used for this purpose. All measurements were conducted in single-use PMMA cuvettes with a path length of 10 mm. All measurements were done at 25 °C because neither the spectrometer could be cooled to 5 °C nor could fibrinogen samples be reasonably measured when stored at this temperature (strongly scattering precipitates and fogging of the cuvette.) For data evaluation, the peak at 437 nm was baseline-corrected, and the corresponding corrected absorbance was used for further interpretation.

To monitor the kinetics, a modified model system was used. The aim is to study fibrinogen concentrations of 0.25, 0.5, 1.0, 1.25, 1.5, and 2.0 g/L in the presence of 15 mmol/L phosphate buffer (pH 7.0) and 15 mmol/L glutaraldehyde.

A fibrinogen stock solution was prepared by suspending 6 mg of fibrinogen in 2 mL of pure water (concentration: 3 g/L) at 25 °C. Then, the pH was adjusted to 7.0 using 0.1 mol/L NaOH. The solution was stirred at 25 °C for 10 min at 300 rpm. To reach the respective fibrinogen concentration, this solution was diluted with water. Then, 15 mmol/L sodium phosphate (pH 7.0) was added. Finally, cross-linking was triggered by adding 15 mmol/L glutaraldehyde from the 500 mmol/L glutaraldehyde stock solution. The reaction mixture was kept in a 25 °C warm water bath. UV/vis measurements were performed after 5, 10, 15, 20, 25, 30, and 60 min and after 24 h. For each set of experiments, i.e., each fibrinogen concentration, a fresh solution was used. The set of experiments was repeated one more time, and the corrected absorbances were averaged.

### 2.6 Deactivating Excess Glutaraldehyde with Lysine

The kinetics of the reaction between glutaraldehyde and lysine was studied by using UV/vis spectroscopy. Details regarding the measurement procedure and data evaluation can be found in Section 2.5.

At first, a 1.5 mol/L solution of lysine hydrochloride was prepared, and its pH was adjusted to 7.0 by using NaOH. For this series of experiments, six times 2 mL of a diluted lysine solution were prepared from the stock solution. These had concentrations of 20, 50, 100, 150, and 200 mmol/L. One additional sample containing 50 mmol/L lysine and 150 mmol/L NaCl was prepared. Then, 60 μL of a 500 mmol/L sodium phosphate buffer (pH 7.0; final concentration: 15 mmol/L) and 60 μL of a 500 mmol/L glutaraldehyde solution were added to each sample (final concentration: 15 mmol/L). Samples were kept in a 25 °C water bath. Measurements were performed after 5, 10, 15, 20, 25, 30, and 60 min and after 24 h. The set of experiments was repeated one more time, and the corrected absorbances were averaged.

### 2.7 Irreversible Dissolution of Pseudofibrin Hydrogels: Scanning Electron Microscopy

The procedure of preparing a SEM sample and the measurement parameters are discussed in Section 2.10.

At first, the protocol for monitoring the thermal dissolution is described. Twenty-four pseudofibrin hydrogels were prepared as described in Section 2.2 using a pH 7.0 sodium phosphate buffer. Afterward, 12 of them were cross-linked by adding 120 μL of a 500 mmol/L glutaraldehyde stock solution (final concentration: 15 mmol/L). The reaction lasted for 10 min at 5 °C. Then, excess glutaraldehyde was deactivated by adding 138 μL of a 1500 mmol/L lysine solution (pH 7.0; final concentration: 50 mmol/L.) This reaction lasted for 30 min at 25 °C. In the meantime (40 min in total), the other 12 gels still rested at 5 °C. Now, the gels (one cross-linked and one non-cross-linked) were exposed to elevated temperatures. The following Table 1 summarizes the applied reaction conditions. For each pair of time and temperature, one cross-linked SEM sample and one non-cross-linked SEM sample were obtained.

**Table 1:** Summary of the Applied Reaction Conditions for Thermal Dissolution of Pseudofibrin Hydrogels^a^

	0.5 h	1 h	2 h	24 h
25 °C				
30 °C				
37 °C				

a Each cell represents one distinct cross-linked and one non-cross-linked sample.

For ion-induced cross-linking, an analogous procedure was applied. Again, 24 gels were prepared as described in Section 2.2 using a pH 7.0 sodium phosphate buffer. Twelve of them were cross-linked as stated above, and also the excess cross-linker was deactivated analogously. The other 12 untreated gels again rested at 5 °C during this time. Then, the ion concentration in all 24 gels was increased by adding defined concentrations of a NaCl stock solution. The final NaCl concentrations were 50, 100, and 140 mmol/L. Again, a summary of the conducted experiments is shown in Table 2.

**Table 2:** Summary of the Applied Reaction Conditions for Ion-Induced Dissolution Pseudofibrin Hydrogels^a^

	0.5 h	1 h	2 h	24 h
50 mmol/L				
100 mmol/L				
140 mmol/L				

a Each cell represents one distinct cross-linked and one non-cross-linked sample.

The NaCl concentrations were reached by adding 160 μL of a 1300, 2600, or 3640 mmol/L NaCl stock solution. All gels were stored at 5 °C during the entire reaction time. For each reaction time and ion concentration, one SEM sample was obtained. In total, 24 samples were taken, two from each pair of time/NaCl concentrations, wherein one gel was cross-linked and the other was not.

### 2.8 Irreversible Dissolution of Pseudofibrin Hydrogels: Dynamic Light Scattering

Dynamic light scattering was performed using a Malvern ZetaSizer Nano-ZS instrument at a scattering angle of 90°. The incident laser had a wavelength of 632.8 nm and a power of 4 mW. Each measured data point is the average of three individual 10 s long measurements. Particle size is expressed as the *z*-average, i.e., assuming a multimodal particle size distribution.

For each measurement, one fibrinogen stock solution was prepared by suspending 2 mg of fibrinogen in 4 mL of pure water (final concentration: 0.5 g/L). Then, the pH was adjusted to 7.0 using 2 μL of a 0.1 mol/L NaOH solution. The mixture was stirred for 10 min at 400 rpm at room temperature. One milliliter of this solution was filtered through a 450 nm polyether sulfone (PES) membrane into a single-use PMMA cuvette. Then, 1 mL of 30 mmol/L sodium phosphate buffer (pH 6.0) was filtered the same way into the solution. The mixture reacted for 4 h at 20 °C. Each reaction mixture was initially characterized for 2.5 min, corresponding to five measurements that lasted 30 s each. Afterward, decomposition was induced.

To monitor thermal decomposition, the temperature in the ZetaSizer was increased to 30 or 37 °C. Immediately afterward, particle sizes were continuously measured for 2 h.

To monitor decomposition induced by increased NaCl concentration, 100 μL of a concentrated NaCl solution was added to each cuvette to reach a desired concentration. An overview of the different experiments and the respective required stock solutions is shown in Table 3. Immediately after adding the respective salt concentration, particle sizes were continuously measured for 2 h.

**Table 3:** Overview of the Three Experiments to Monitor Ion-Induced Decomposition of Pseudofibrin^a^

final NaCl concentration	added volume of the NaCl stock solution	concentration of NaCl stock solution
50 mmol/L	100 μL	1050 mmol/L
100 mmol/L	100 μL	2100 mmol/L
140 mmol/L	100 μL	2940 mmol/L

a Shown are the final NaCl concentrations in the cuvette and concentrations of the respective NaCl stock solution.

### 2.9 Gravimetric Measurements of Dried Hydrogels

Ten pseudofibrin hydrogels were prepared as described in Section 2.2 using a pH 7.0 sodium phosphate buffer. After the usual 4 h reaction time, all gels were cross-linked with 120 μL of a 500 mmol/L glutaraldehyde solution (final concentration: 15 mmol/L) for 10 min at 5 °C. Then, excess glutaraldehyde was deactivated by adding 138 μL of a 1500 mmol/L lysine solution (pH 7.0; final concentration: 50 mmol/L). This reaction lasted 30 min at 25 °C. The gels were then exposed to elevated temperatures. The temperature program and the respective number of gels are listed in Table 4.

**Table 4:** Summary of the Applied Temperature Programs to Study the Thermal Dissolution of Pseudofibrin Hydrogels Gravimetrically

temperature	time	number of gels	remarks
5 °C	24 h	2	references
25 °C	24 h	2	
30 °C	24 h	3	
37 °C	24 h	3	

Afterward, all gels were carefully extracted from the reaction vessel. They were weighed wet, dried overnight at room temperature, and weighed again. In the dry sample, not only the pseudofibrin gel is weighed but also crystallized salts. Their mass was calculated by the amount of water, which is why the sample was also weighed wet. Because the mass (and therefore the volume) of residual water is known, the amount of salt in this residual water can be calculated. The masses of the dried gels were corrected accordingly and averaged.

### 2.10 Preparation of Samples for Scanning Electron Microscopy

Scanning electron microscopy (SEM) was performed using a Zeiss Neon 40. Topography images were obtained by using an SE2 detector, and material contrast was monitored by an InLens detector. An acceleration voltage of 2 kV was applied.

Before each measurement, samples were coated with a 3 nm thin layer of a Pd/Au alloy by using a BalTec SDC 500 Sputter Coater.

To obtain a SEM sample, a clean microscopy cover slide (12 × 12 mm) was held in the reaction mixture/the gel for 5 min. This was done at room temperature. Afterward, the glass slide was carefully removed and dried at room temperature. On a side note, an alternative way of preparing the samples is by lyophilization. However, this does not preserve the fibrous structures well. This makes it impossible to scientifically study any changes/damages to the fibers due to the cross-linking procedure.

## 3 Results and Discussion

### 3.1 Initial Situation and Work Plan

**Figure 1:**
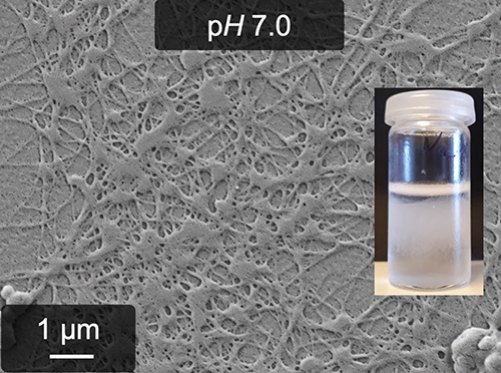
Photograph of the initial, non-cross-linked pseudofibrin hydrogel and a SEM image showing its fibrous structure.

Recently, we presented several approaches to create a fibrin-like hydrogel from fibrinogen by adding defined salts under suitable reaction conditions. Due to its similarity to actual fibrin, this material is labeled “pseudofibrin”. One fabrication route relies on the addition of kosmotropic anions, for example, a sodium phosphate buffer, to an otherwise salt-free solution. In particular, the fibrinogen stock solution has to be at its solubility limit, which means a temperature of 5 °C, a pH of 7.0, and a sodium phosphate concentration of 15 mmol/L. If these conditions are not overstepped, adding the phosphate buffer to the cold fibrinogen solution leads to a soft, gel-like material during 4 h at 5 °C. For a better overview, a photograph of the gel-like material and a SEM image of its fibrous structure are shown in Figure 1. This image, as well as further details regarding this material, can be found in one of our earlier works.^28^

**Figure 2:**
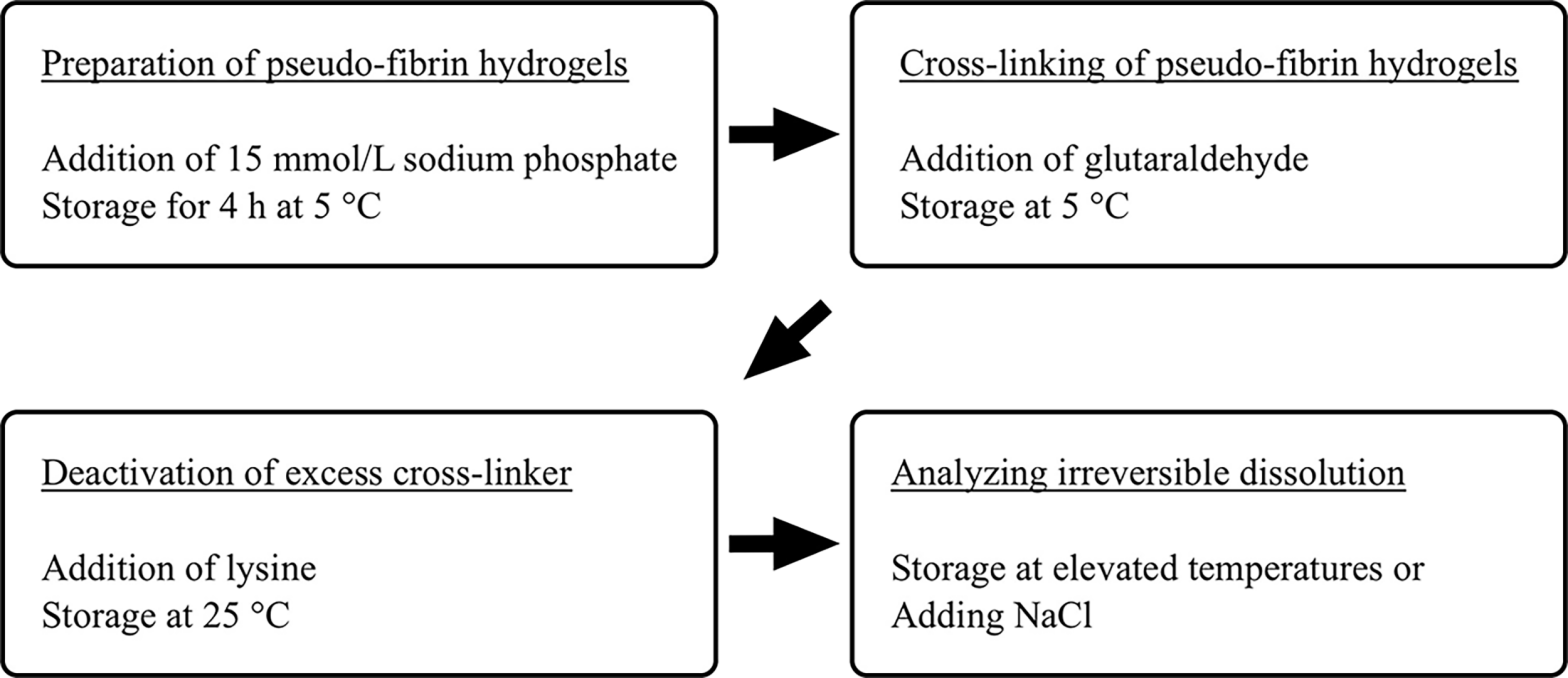
Schematic representation of the anticipated workflow. At first, pseudofibrin hydrogels were prepared. In the next step, these were covalently stabilized with glutaraldehyde. The excess glutaraldehyde was then removed. Finally, the effects of cross-linking on irreversible dissolution shall be studied.

This type of pseudofibrin, however, would only have very limited applicability because the gels are mechanically rather instable. Additionally, it is already known from previous experiments that the fabricated gels are sensitive to elevated temperatures, leading to rapid dissolution. To improve the material properties, the gels would benefit from covalent cross-linking. Due to their mechanical and thermal instability, cross-linking will initially be performed at 5 °C by adding the desired amount of glutaraldehyde to the “floating” gel. In the following steps, excess glutaraldehyde must be deactivated, preferably with a primary amine. A schematic representation of the anticipated procedure is shown in Figure 2.

### 3.2 Identification of Optimal Cross-Linking Conditions

**Figure 3:**
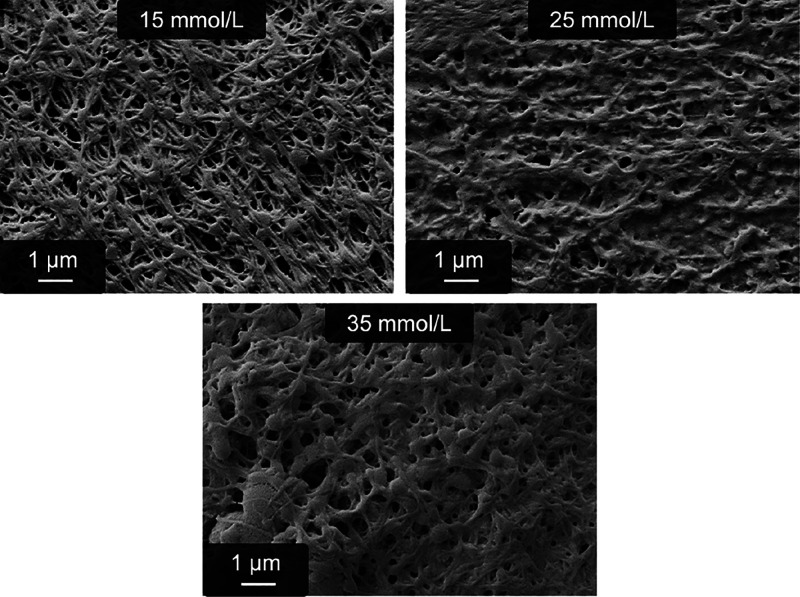
Scanning electron microscopy images of glutaraldehyde cross-linked pseudofibrin fibers as extracted from the reaction vessel. Clearly, glutaraldehyde concentrations greater than 15 mmol/L significantly damage the fibrous structure and lead to a clot of unstructured proteins.

At first, a suitable glutaraldehyde concentration was identified. It has to be considered that glutaraldehyde exhibits a highly complex behavior in aqueous solution, which includes, for example, oligomers of varying chain lengths and intramolecular cyclizations. For this reason, the actual concentration of reactive groups is unknown but significantly lower than 2 times the used glutaraldehyde concentration.^42^ For comparison, the used 5 g/L fibrinogen contains in sum 3.1 mmol/L lysine that can react with glutaraldehyde.^45^ To get an impression of how fast and effective the gels can be stabilized at all, the highest glutaraldehyde concentration possible shall be identified. This concentration should be high enough to enable fast kinetics at 5 °C while at the same time retaining the fibrous structure of the material. Especially an aggregation to an unstructured clot must be prevented. Concentrations of 15, 25, and 35 mmol/L were applied for 15 min. Already after several minutes (even at 5 °C), a characteristic yellow/brown coloration emerged, indicating a fast cross-linking reaction. This coloration is typical for the reaction of glutaraldehyde and amines and most likely stems from the formation of a Schiff base.^46^ Again, due to the complex chemistry of glutaraldehyde, the exact chemical nature of the reaction products is unpredictable.^42^ Scanning electron microscopy images of the fibers are shown in Figure 3. It can be seen that the fibrous structure is retained at 15 mmol/L glutaraldehyde, whereas 25 mmol/L already leads to a loss of the fibrous structures. Instead, a rather undefined clot is observed. This effect is even more pronounced at 35 mmol/L glutaraldehyde. Remarkably, all three SEM images show incorporated “spheres”. Their origin is currently unknown but may be related to the initial precipitation of fibrinogen upon phosphate addition.^28^ For the following experiments, 15 mmol/L glutaraldehyde is set as the reference concentration.

**Figure 4:**
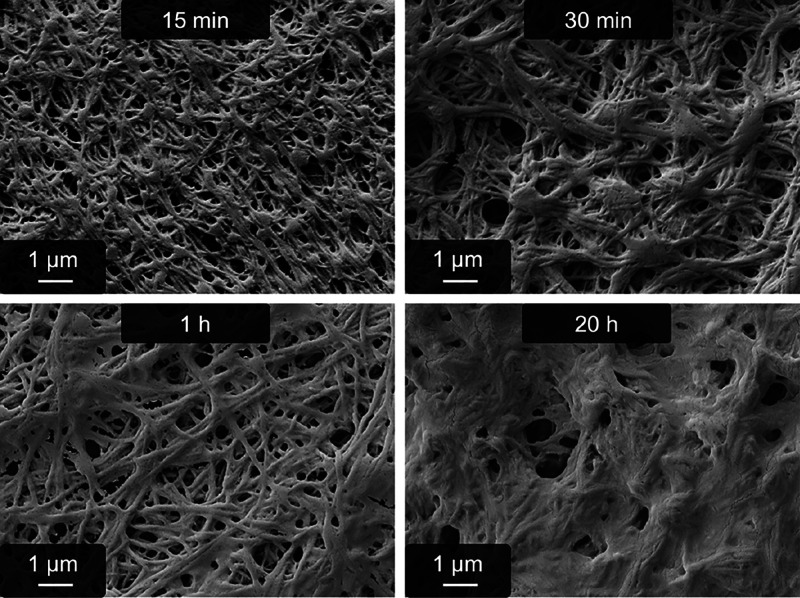
SEM images of glutaraldehyde cross-linked pseudofibrin fibers after different reaction times. The fibrous structure is still intact after 1 h of reaction time, whereas only an unstructured protein clot remains after 20 h.

The next step is to identify a possible time frame for successful cross-linking. Again, the main priority is to retain the fibrous structure. Glutaraldehyde was allowed to react for 15 min, 30 min, 1 h, and 20 h with pseudofibrin. The corresponding SEM images are shown in Figure 4. The fibers are intact for at least up to 1 h reaction time and (qualitatively) became more resistant against mechanical damage. This already became obvious, as the gel could be extracted from the vessel in one piece instead of many small fragments. More quantitative insights are given in the following sections. Again, spherical structures incorporated into the gel can be found. The images prove that there is a comparatively broad time frame of approximately 1 h of reaction time in which cross-linking can be performed. This potentially even allows tailor-made mechanical properties by simply adjusting the reaction time. This goes, however, beyond the scope of this work.

Although a suitable cross-linking protocol is now identified (15 mmol/L glutaraldehyde, <60 min reaction time at 5 °C), one additional experiment was done regarding the cross-linking temperature, which is currently set to 5 °C. An increased reaction temperature of 25 °C, however, did not lead to any fibrous structures. The reaction rate of glutaraldehyde was too high, which resulted in the known unstructured clot. Further, partial dissolution of the pseudofibrin gels at early stages of cross-linking cannot be excluded either (see Section 3.6.) For this reason, regarding the fiber morphology, the optimal reaction conditions for glutaraldehyde with pseudofibrin are a temperature of 5 °C, a glutaraldehyde concentration of 15 mmol/L, and a reaction time of 60 min at most.

### 3.3 Mechanical Properties of Pseudofibrin Gels before and after Cross-Linking

**Figure 5:**
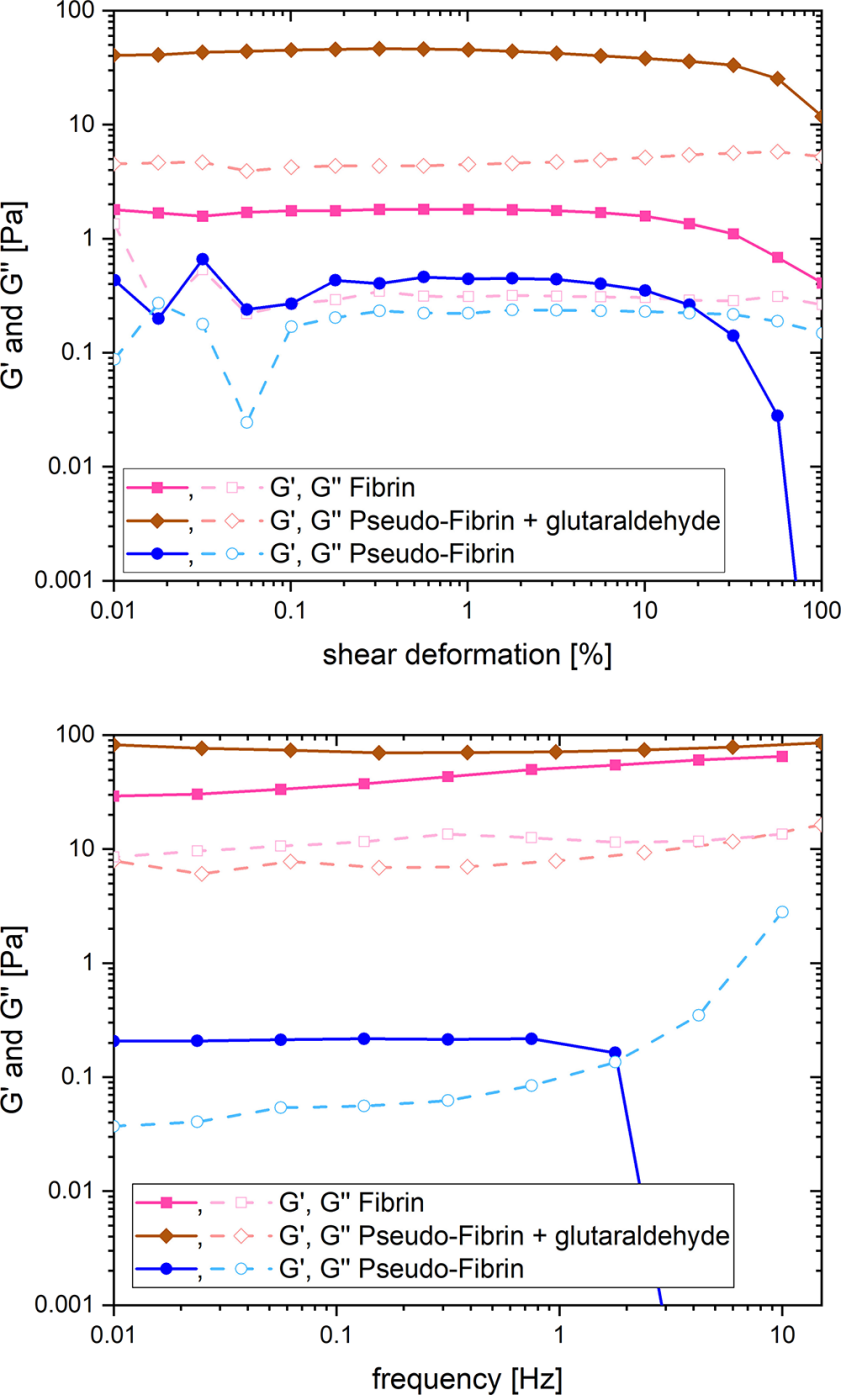
Amplitude (top) and frequency (bottom) scan of pseudofibrin (○), pseudofibrin cross-linked with glutaraldehyde (◊), and noncovalently cross-linked fibrin (■). Open symbols represent *G*′; closed symbols represent *G″*.

After qualitatively identifying optimal cross-linking conditions, which predominantly retain fiber morphology, the next step is to quantify the effect of additional covalent bonds in the system. A suitable and frequently applied method for this purpose is rheological measurement.^47,48^ To characterize covalently cross-linked gels, especially in comparison to a non-cross-linked sample, amplitude and frequency scans were performed to monitor the evolution of storage modulus *G*′ and loss modulus *G″*. The results are listed in Figure 5.

The non-cross-linked reference overall shows low moduli <1 Pa, proving that the material is very soft. In general, high moduli indicate high stiffness, mostly resulting from a high degrees of cross-linking.^48,49^ The increase in moduli after cross-linking therefore shows that the gels are indeed stabilized, confirming the optical impression. Glutaraldehyde cross-linked gels exhibit a *G*′ of approximately 40 Pa, which is still rather low but 200-fold that of the non-cross-linked reference.

After cross-linking, the linear viscoelastic regime, i.e., the plateau in the amplitude scan, is expanded. The (potential) crossover between *G*′ and *G*″ is not even in the measured range anymore. A decrease of *G*′ and *G″* is indicative of damages of the material, for example, in the form of breaking noncovalent bonds or separation of protein and water. The fact that this effect now requires significantly higher shear deformations is a major improvement in material properties.

Similar information can be obtained from the frequency scans. These especially indicate the reversibility of cross-linking in form of an intersection of *G*′ and *G*′′.^48,49^ Non-cross-linked pseudofibrin hydrogels show such intersection at 2 Hz, whereas no intersection is observed within the measured range for the covalently cross-linked sample. In a frequency scan, an increase of *G*′ and *G*″ is expected at higher frequencies. This, however, cannot be observed for untreated pseudofibrin, presumably because the gel structure already collapses at frequencies >1 Hz.

A relevant comparison in this context is how noncovalently cross-linked fibrin performs under comparable conditions. Previously, we measured a storage modulus of 20–30 Pa in the frequency scan.^28^ The cost-efficient nonenzymatic fabrication route of pseudofibrin might still be the most relevant advantage compared to fibrin. Because the storage modulus of glutaraldehyde cross-linked gels is approximately double that of fibrin, this approach might be attractive for future applications. As seen in Section 3.2, glutaraldehyde cross-linking retains the fibrous structure up to a reaction time of 1 h, so it is reasonable to assume that mechanical properties of pseudofibrin can be tailored even further. Although this would also be possible with fibrin, the great advantage of pseudofibrin is its cost-efficiency, and the rheologic measurements show that it is in principle possible to mimic fibrin in terms of morphology and mechanical properties.

### 3.4 Estimation of Cross-Linking Kinetics and Degree of Conversion

**Figure 6:**
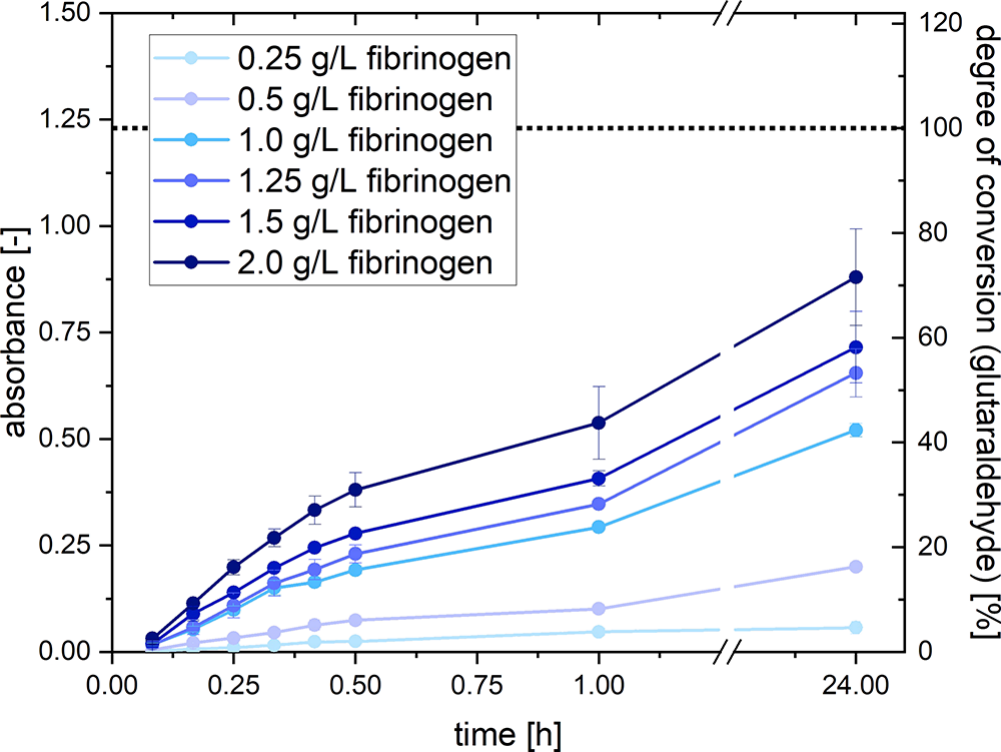
Time-dependent absorbance at 437 nm of a fibrinogen/glutaraldehyde mixture. Absorbance of 1.23 marks 100% conversion of glutaraldehyde.

Based on the results up to this point, glutaraldehyde cross-linking qualitatively and quantitatively yields promising results for pseudofibrin stabilization. As a next step, cross-linking kinetics will be estimated. Because the reaction of glutaraldehyde and amines leads to yellow-brown products, the progress of the reaction can, in principle, be followed by UV/vis spectroscopy. This, however, only works for comparatively diluted fibrinogen solutions, as the cross-linked proteins strongly scatter the incident laser beam. For the same reason, cross-linking of pseudofibrin gels as such cannot be followed. As a model system, native fibrinogen in phosphate buffer was chosen and cross-linked with the usual amount (15 mmol/L) of glutaraldehyde. This was done for fibrinogen concentrations from 0.25 to 2.0 g/L. For data evaluation, the peak at 437 nm (corresponding to the yellowish coloration) was used. For reference, Figure S1 in the Supporting Information shows a non-baseline-corrected UV/vis spectrum of lysine and glutaraldehyde. The results are shown in Figure 6.

**Figure 7:**
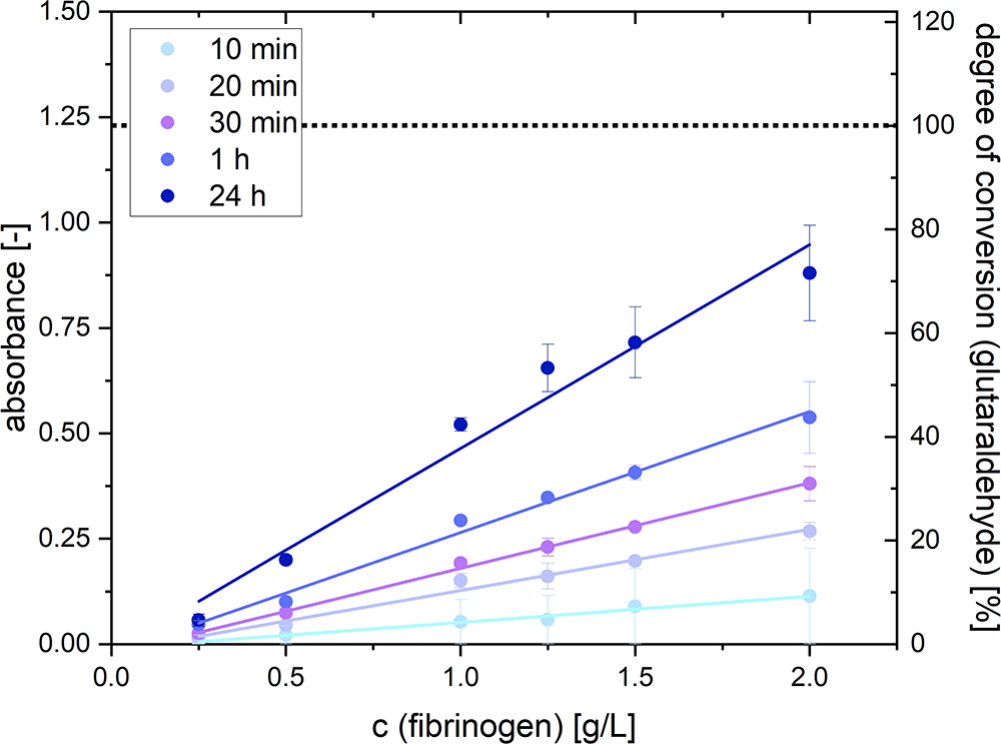
Concentration-dependent absorbance at 437 nm of a fibrinogen/glutaraldehyde mixture at different reaction times. An absorbance of 1.23 marks 100% conversion of glutaraldehyde.

Obviously, the coloration of the mixture becomes stronger when higher fibrinogen concentrations and long reaction times are applied. In all cases, the cross-linking kinetics is fastest during the first hour. At low fibrinogen concentrations (0.25 g/L), the degree of conversion of amine groups appears to be nearly 100% after 1 h because the absorbance reaches a plateau. This cannot be observed for other concentrations. Practically, however, the kinetics during the first hour is the main interest here because this is exactly the time frame in which the fibrous structures of pseudofibrin remain undamaged (see Section 3.2). The diagram also contains a dashed line at an absorbance of 1.23. This marks 100% conversion of 15 mmol/L glutaraldehyde. The exact origin of this value will be elucidated later in the context of the deactivation of glutaraldehyde with lysine. It, however, reveals one first interesting aspect about the kinetics: the fact that fibrinogen is the limiting factor in this case. Otherwise, all curves would have reached a maximum absorbance of 1.23. Additionally, it allows us to estimate how much of the glutaraldehyde reacted with fibrinogen after a distinct time. Because the data cannot be fitted with kinetics of first, pseudo-first, second, or third order (potentially due to the complex chemistry of glutaraldehyde^42^), another approach has to be chosen. For this purpose, the fibrinogen concentration is plotted against absorbance, as shown in Figure 7. Here, the graphs represent different reaction times. A summary of the extracted data can be found in Table 5.

**Table 5:** Details of the Concentration-Dependent Absorbance of a Fibrinogen/Glutaraldehyde Mixture (Figure 7)^a^

**time**	**slope**	**intercept**	***R***^**2**^
10 min	0.0618 ± 0.0041	–0.0100 ± 0.0051	98.26
20 min	0.1450 ± 0.0088	–0.0175 ± 0.0109	98.53
30 min	0.2030 ± 0.0949	–0.0234 ± 0.0060	99.77
1 h	0.2869 ± 0.0136	–0.0219 ± 0.0136	99.11
24 h	0.4832 ± 0.0428	–0.0188 ± 0.0428	96.96

a Given are reaction times and the respective parameters for linear regression.

As can be seen, the graphs corresponding to reaction times of 10–60 min can be fitted with a linear function. Only the values at 24 h show a more exponential increase. One reason, especially at higher concentrations, might be the strong scattering stemming from the cross-linked aggregates, which would shift the measured absorbances to lower values.

Assuming an ongoing linear trend at reaction times from 10 to 60 min, the graphs can be extrapolated to estimate how much glutaraldehyde actually reacted with fibrinogen in the scenarios presented in Section 3.2. For example, cross-linking 5 g/L fibrinogen for 10 min theoretically yields an absorbance of approximately 0.3, corresponding to a conversion of 24%. It is important to remember that this is only a theoretical approximation that becomes increasingly unprecise at higher reaction times, as can already be seen at the curve corresponding to 24 h reaction time. Additionally, it must be considered that not all fibrinogen is incorporated into the fibrous structures. Approximately 80% of fibrinogen is still in the solution (see also Section 3.6 and especially Figure 12), which makes it impossible to determine which fraction of the reacted glutaraldehyde actually leads to cross-linking of the fibers. For this reason, only an estimation of the overall degree of glutaraldehyde conversion can be given. In the actual experimental setup, the reaction kinetics of glutaraldehyde and pseudofibrin fibers will most likely involve complex transport processes including diffusion from the surface to the central regions.

One final piece to estimate the degree of conversion is still missing: the influence of temperature. To prevent precipitation, these experiments were done at 25 °C, while the actual cross-linking reaction of pseudofibrin takes place at 5 °C to not dissolve the gels (see also Section 3.6 for more insights into thermal dissolution). As a rule of thumb, the reaction rate halves when lowering the temperature by 10 °C. In this case, this would correspond to an approximate degree of conversion of 6% for a reaction time of 10 min. Although decreased kinetics might favor the applicability of the model, many assumptions and extrapolations had to be made to obtain this value. It therefore represents a reasonable estimation rather than a reliable value.

Further, it has to be noted that glutaraldehyde forms various oligomers depending on the pH, so the actual concentration of functional groups is never 15 mmol/L.^42^ An exact concentration cannot be given, only the estimation that a low amount of these will react with fibrinogen. This raises another issue, i.e., the large amounts of excess glutaraldehyde. Not only will these continue to cross-link pseudofibrin and damage its fibrous structure, but they are also harmful. Especially for applications of pseudofibrin, this excess of glutaraldehyde must be removed. Solving this issue is the subject of the following section.

### 3.5 Deactivating Excess Glutaraldehyde with Lysine

In the previous section, it could be estimated that only low amounts of glutaraldehyde actually react with pseudofibrin during cross-linking. Otherwise, the fibrous structure will be damaged, and the result is an unstructured protein clot. To prevent the excess glutaraldehyde from further reactions, be it with fibrinogen or with organic tissue that comes in contact with the gel, a suitable method has to be developed to quench the reaction. For aerogels, lyophilization would be a promising method to evaporate glutaraldehyde. To retain the hydrogels, another approach has to be chosen. Because glutaraldehyde readily reacts with primary amines,^41^ addition of lysine appears to be a reasonable deactivation agent. This strategy can also be found in other literature reports.^40^ In a later stage, the byproducts must be removed from the gel, but this study focuses on a proof-of-principle for the deactivation step.

**Figure 8:**
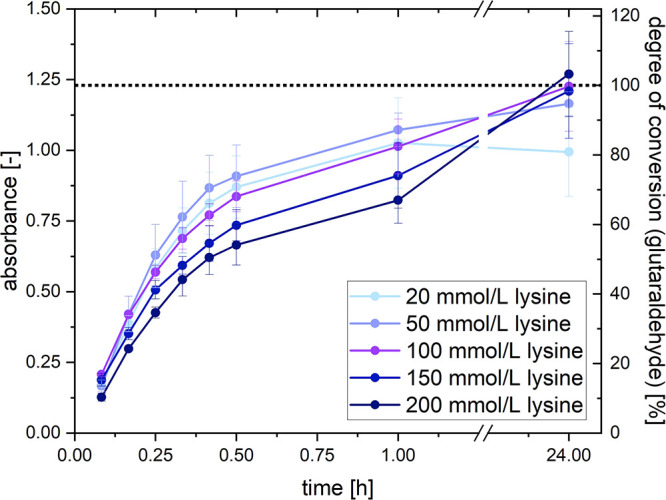
Time-dependent absorbance at 437 nm of a lysine/glutaraldehyde mixture. An absorbance of 1.23 marks 100% conversion of glutaraldehyde.

There are two main parameters that must be adjusted. These are the lysine concentration and reaction time. Both should be as low as possible to reduce the amount of contaminants in the gel and to quench the reaction without delay. Both questions were again studied by using UV/vis measurements. This time, different concentrations of lysine were combined with the usual concentration of 15 mmol/L glutaraldehyde. The reaction lasted for 24 h, and absorbance as an indicator for reaction progress was measured. The results are visualized in Figure 8.

This diagram reveals several remarkable aspects about the reaction of interest. First, it can be seen that the curves of 100, 150, and 200 mmol/L lysine all reach nearly the same absorbance after 24 h. This effect can be seen even better in Figure S2 in the Supporting Information. This indicates that all functional groups of glutaraldehyde have reacted with lysine, hence the same absorbance in all cases. The averaged absorbance in all three cases is 1.23, which was therefore assumed to represent 100% conversion of glutaraldehyde. This value was already used in Section 3.4.

Second, the reaction of lysine with glutaraldehyde mainly happens in the first 30 min after their combination. For the actual purpose of cross-linking, this is ideal: Because pseudofibrin’s fibers are undamaged until 1 h of cross-linking, glutaraldehyde can be incubated for approximately 10 min. Then, there is enough time left to deactivate (most) of the excess glutaraldehyde.

The third observation was not expected. The reaction rate apparently shows a maximum at approximately 50 mmol/L lysine. Higher amounts lower the rate so that 200 mmol/L lysine reacts significantly slower than 20 mmol/L. The explanation is that lysine was used in the form of its monohydrochloride for solubility reasons. The additional chloride ions, however, decelerate the reaction rate between lysine and glutaraldehyde. For comparison, Figure S3 in the Supporting Information compares the reaction of 15 mmol/L glutaraldehyde with 50 mmol/L lysine, 200 mmol/L lysine, and 50 mmol/L lysine combined with 150 mmol/L NaCl.

In summary, the addition of 50 mmol/L lysine is a promising way to quench the cross-linking of fibrinogen. Based on the absorption values, incubation of 50 mmol/L lysine for 30 min will deactivate approximately 74% of the free glutaraldehyde, while the reaction between fibrinogen and glutaraldehyde already consumes approximately 6% glutaraldehyde. The final section shall now reveal how (irreversible) dissolution of pseudofibrin hydrogels is affected by cross-linking and how the developed route of cross-linking and deactivation alters the microscopic structure of the gels.

### 3.6 Thermal Dissolution of Pseudofibrin Hydrogels

**Figure 9:**
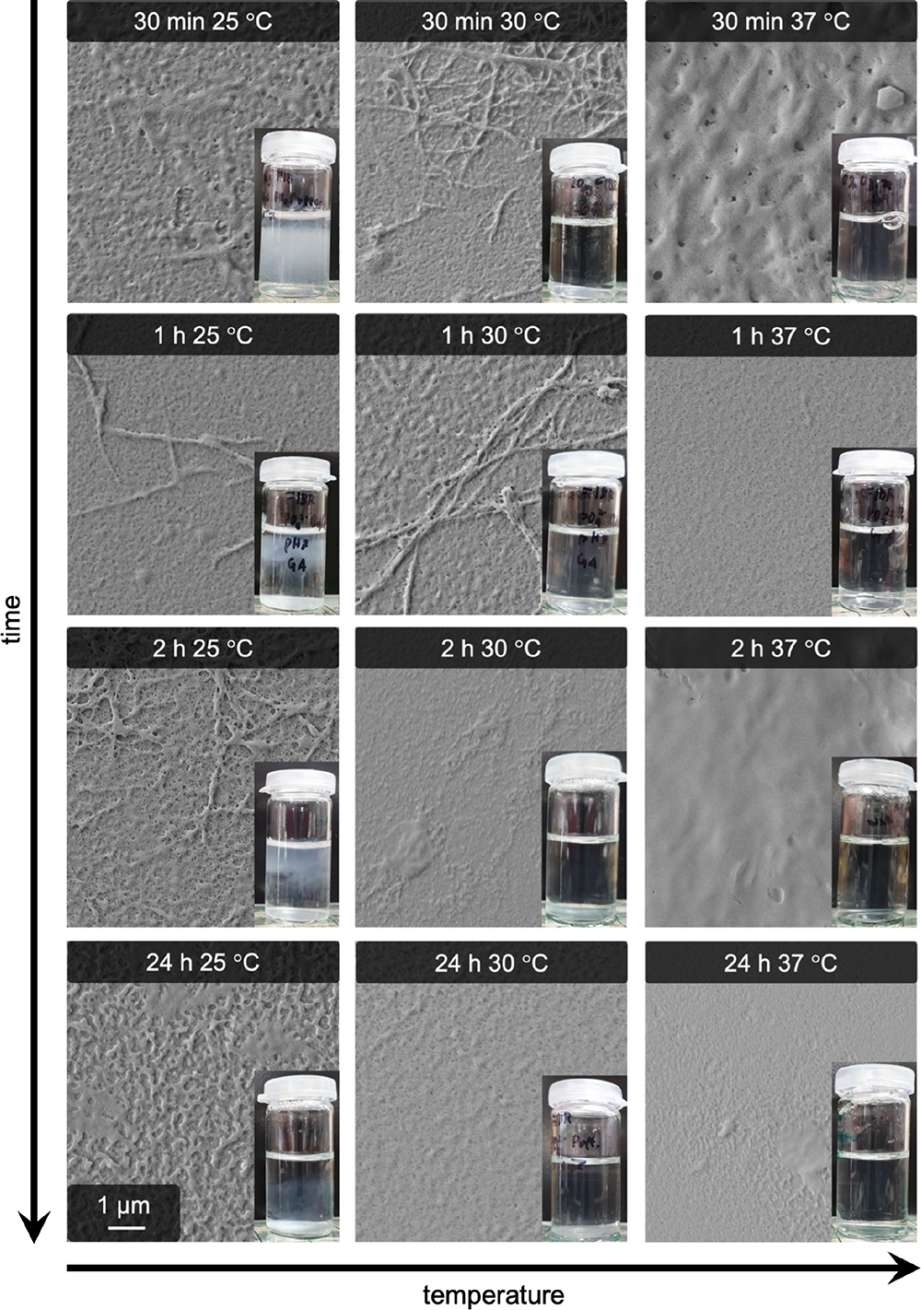
Effect of elevated temperatures (25, 30, and 37 °C) on pseudofibrin hydrogels and the remaining fibers. The higher the temperature is, the faster the gels dissolve. Storage for 30 min at 30 °C is already sufficient to dissolve nearly the whole gel and fibers.

As stated in one of our earlier publications regarding pseudofibrin,^28^ optimal reaction conditions to create hydrogels via addition of kosmotropic anions are close to fibrinogen’s solubility limit. Adding the respective salt under physiological conditions leads to neither fibrillogenesis nor gelation. Actually, these pseudofibrin gels irreversibly dissolve when exposed to conditions closer to physiological ones. The most pronounced effect was observed for elevated temperatures, which lead to dissolution of pseudofibrin within minutes. The qualitative evolution of pseudofibrin dissolution at 25, 30 and 37 °C is shown in Figure 9. As can be seen, the gels already dissolved at room temperature. Notably, the “cloudy” impression of the gels does not imply fiber formation as most SEM images taken after storage at 25 °C only show a few residuals of fibers. The higher the temperature is and the longer this temperature is applied, the more gels and fibers dissolve.

**Figure 10:**
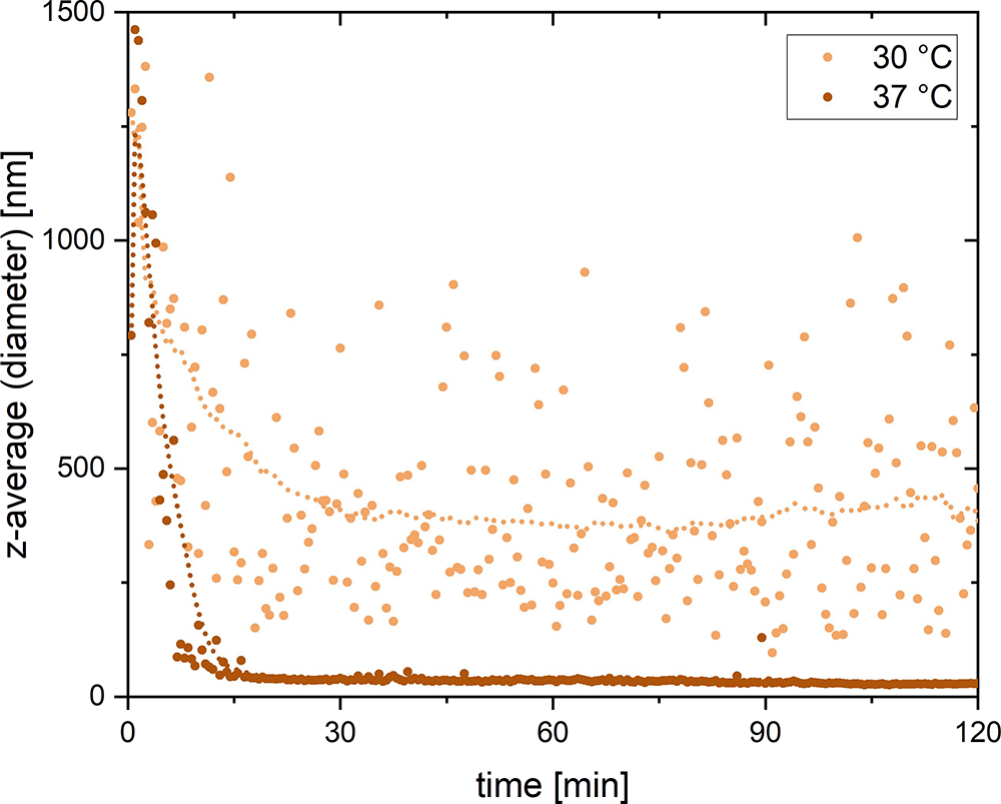
Thermal dissolution of pseudofibrin studied by means of dynamic light scattering. Evolution of the *z*-averaged particle diameter during the first 2 h of tempering is shown. The graphs were recorded at temperatures of 30 and 37 °C. Dotted lines represent the average particle size.

To obtain a more quantitative insight into pseudofibrin dissolution, dynamic light scattering was performed. Gels were first prepared at a lower concentration to reduce the scattering signal and then tempered in the device for 2 h. The results are expressed as the *z*-average, i.e., the average particle diameter if a monodisperse solution is assumed. Additionally, the average particle size at a given time is shown. Figure 10 expectedly shows that especially a temperature of 37 °C leads to complete dissolution of the gels within 15–20 min, which is in accordance with the SEM images in Figure 9. The data points of the second graph recorded at 30 °C strongly fluctuate, indicating the presence of several larger aggregates. Again, SEM images show the presence of fibers until at least 1 h reaction time. Apparently, the reaction mixture still contains several aggregates after 2 h reaction time, which could however not be identified optically or via SEM. Due to the large particle size, a light scattering measurement at 25 °C (and below) was not reasonable.

To investigate whether covalent cross-linking can prevent the dissolution of pseudofibrin gels, the experiments were repeated with glutaraldehyde cross-linked gels. For cross-linking, the previously elaborated protocol was applied. Briefly, the steps to study thermal dissolution were:

- Preparation of pseudofibrin gels via addition of sodium phosphate
- Cross-linking with glutaraldehyde (10 min)
- Deactivation of glutaraldehyde with lysine (30 min)
- Exposure of gels to elevated temperatures

**Figure 11:**
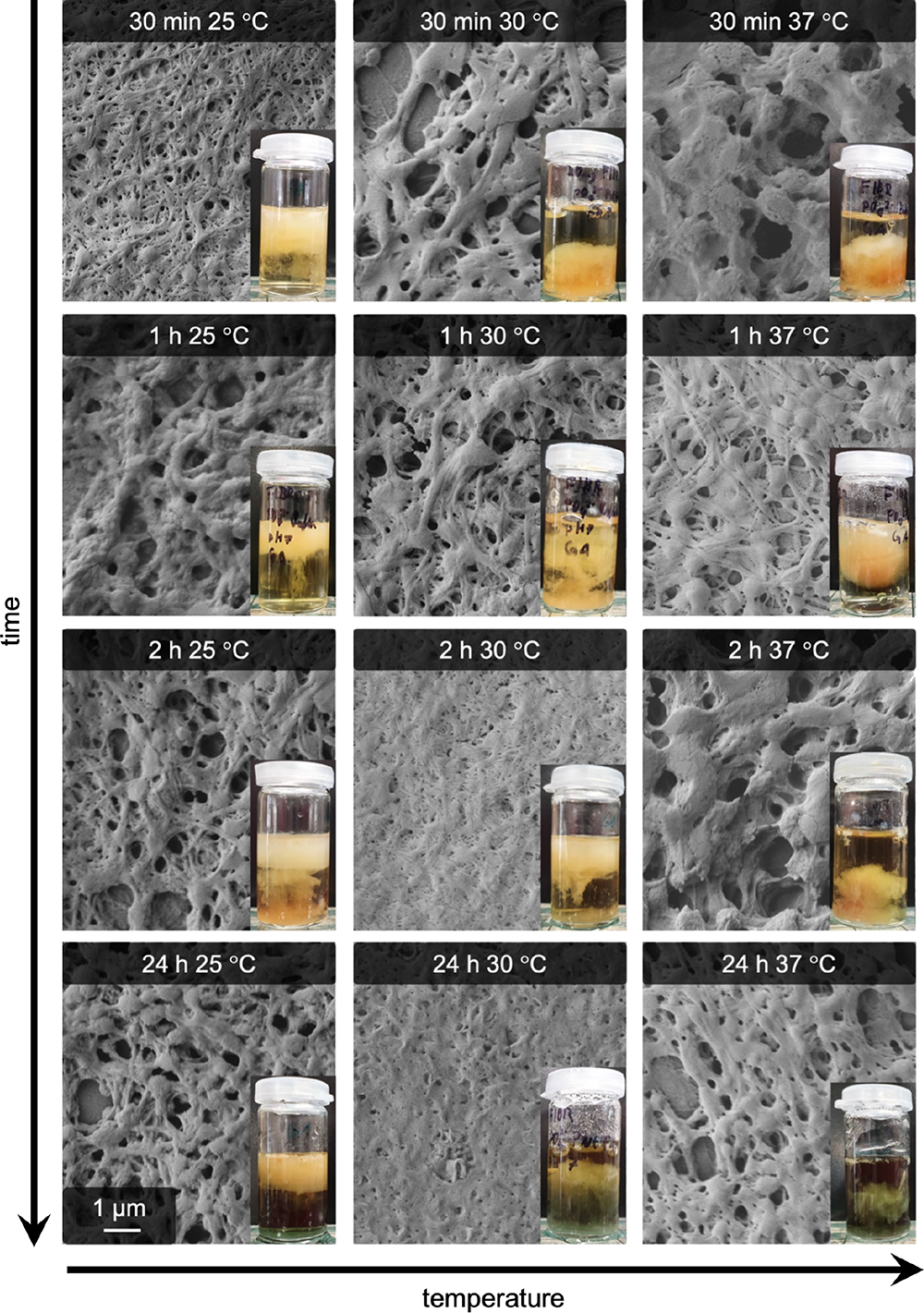
Effect of elevated temperatures (25, 30, and 37 °C) on glutaraldehyde cross-linked pseudofibrin hydrogels and the remaining fibers. In all cases, the fibrous structure is retained, although especially samples at 37 °C and long exposure times show a less structured morphology.

The results are listed in Figure 11. Based on the optical impression of the hydrogels, cross-linking apparently prevented the dissolution of the gels. However, microscopy images show that the fibers are less defined than the untreated references. The structures seen on all samples vary and show no clear trend. In summary, the samples appear not to be significantly different from each other, meaning that the applied temperature program indeed does not affect the gels anymore.

**Figure 12:**
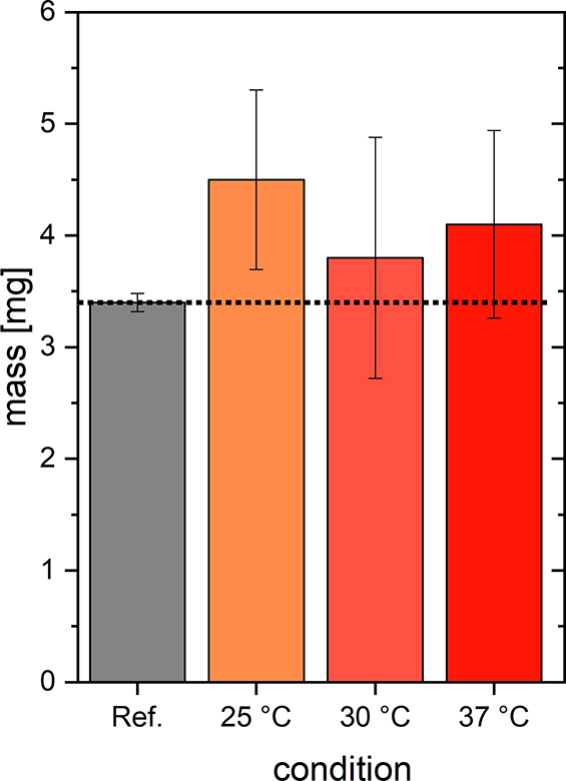
Weight-loss of cross-linked pseudofibrin hydrogels after 24 h at 25, 30, and 37 °C compared to a cross-linked but otherwise untreated reference.

Still, quantitative insight into the dissolution of cross-linked fibers is missing. Dynamic light scattering is not possible anymore due to the larger particle size. For this reason, the degree of dissolution is determined gravimetrically. Cross-linked pseudofibrin samples were stored for 24 h in 25, 30, or 37 °C warm buffer, dried, and weighed. As a reference, a cross-linked but otherwise untreated sample was used. The results are shown in Figure 12. After drying, all samples, including the reference, weigh approximately 3–5 mg, corresponding to 15–25% of the originally used protein mass (20 mg/4 mL). Within significance, all tempered hydrogels had the same mass. Remarkably, the mass of the gel stored at 25 °C appears to be slightly higher than that of the reference. This finding might be attributed to the sample preparation because there is no plausible explanation of why the hydrogel mass should increase specifically at this temperature. Still, the main purpose of these experiments was to study whether a weight loss can be detected upon tempering the cross-linked samples, which is definitively not the case. Glutaraldehyde cross-linking is therefore a suitable tool not only to enhance mechanical properties of the gels but also to prevent their irreversible dissolution. Especially for actual applications, this resembles a major improvement of the original material.

On a side note, elevated ion concentrations would also lead to partial dissolution of pseudofibrin. This effect is, however, significantly less pronounced, which is why this work focuses on thermal dissolution. Time-resolved measurements of particle sizes in analogy to Figure 10 and electron microscopy images of the corresponding gels in analogy to Figures 9 and 11 are shown in the Supporting Information (Figures S4–S6).

## 4 Conclusions

In this work, covalent cross-linking of phosphate-induced pseudofibrin hydrogels was investigated. As a model system, glutaraldehyde was chosen due to its fast reaction rate and its general popularity among multiple literature reports. The optimal, i.e., highest possible, glutaraldehyde concentration to not damage the fibrous structure is 15 mmol/L, while the reaction itself can last at least 1 h without any damages. By applying this protocol, the stability of the hydrogels can be significantly increased; within 10 min, the storage modulus of the gel increases by approximately 200-fold to 40 Pa. Additionally, the cross-linking step prevents the thermally induced dissolution of the gels. Quantitative insights into pseudofibrin cross-linking were obtained by UV/vis measurements because the reaction of proteins (i.e., primary amines/lysine) with glutaraldehyde leads to a characteristic yellow/brown coloration with a maximum absorbance at 437 nm. Besides valuable knowledge about the general cross-linking kinetics of fibrinogen with glutaraldehyde, these measurements allowed estimation of the degree of conversion for the given system, which is approximately 6%. These measurements were accompanied by UV/vis measurements of the reaction of glutaraldehyde with pure lysine. These should give an idea of whether addition of lysine to the cross-linked hydrogel is a suitable method to quench the reaction and deactivate excess glutaraldehyde. Kinetic measurements showed that addition of 50 mmol/L lysine hydrochloride deactivates approximately 70% of glutaraldehyde within 30 min. Higher concentrations were not reasonable because the increased ion concentrations (again, lysine hydrochloride is used) decrease the reaction rate with glutaraldehyde.

Upcoming research will address the question of how mechanical properties can be tailored by choosing different reaction times and other cross-linking agents like genipin and formaldehyde. Additionally, purification of the cross-linked and quenched hydrogels will be studied in more detail.

## Supporting Information Available

UV/vis spectra of a glutaraldehyde/lysine mixture after 0 and 30 min (Figure S1); absorbance of different glutaraldehyde/lysine mixtures vs lysine concentration (Figure S2); time-dependent absorbance at 437 nm of 15 mmol/L glutaraldehyde with 50 mmol/L lysine, 200 mmol/L lysine, and 50 mmol/L lysine combined with 150 mmol/L NaCl (Figure S3); DLS measurements of NaCl-induced dissolution of pseudofibrin during the first 2 h after addition of NaCl (Figure S4); SEM images showing the effect of increased NaCl concentrations (50, 100, and 140 mmol/L) on pseudofibrin hydrogels and the remaining fibers (Figure S5); and SEM images showing the effect of increased NaCl concentrations (50, 100, and 140 mmol/L) on glutaraldehyde cross-linked pseudofibrin hydrogels and the remaining fibers (PDF)
