# Supplementary material for: Glutaraldehyde Cross-Linking of Salt-Induced Fibrinogen Hydrogels
Source: ACS Biomater Sci Eng. 2024 Oct 18;10(11):6927–37. doi: 10.1021/acsbiomaterials.4c01412 (PMC11558561; doi:10.1021/acsbiomaterials.4c01412)
Supplement: Supplementary file 1 — ab4c01412_si_001.pdf [file ab4c01412_si_001.pdf]

Glutaraldehyde Cross-Linking of Salt-Induced Fibrinogen Hydrogels

*Dominik Hense[1] and Oliver I. Strube[1]**

[1] Institute for Chemical Engineering, University of Innsbruck, Innrain 80-82, 6020 Innsbruck, AT

* corresponding author: oliver.strube@uibk.ac.at, phone: +43 512 507 55300

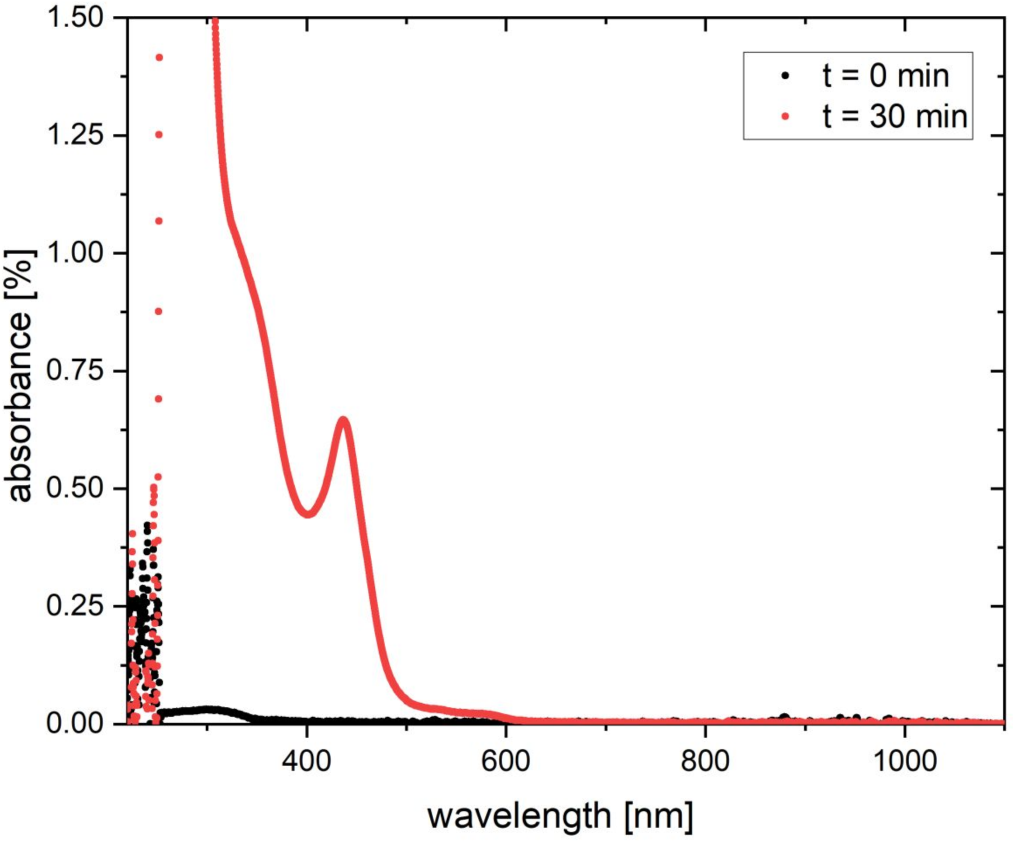

Figure S1. UV/VIS spectrum of 15 mmol/L glutaraldehyde + 50 mmol/L lysine hydrochloride at t = 0 and t = 30 min. The spectra are not yet baseline-corrected. The peak at 250 nm resembles protein aggregates, the peak at 437 nm emerges during cross-linking. The reaction of glutaraldehyde and amines yields a characteristic yellow/brown product due to formation of a Schiff base.

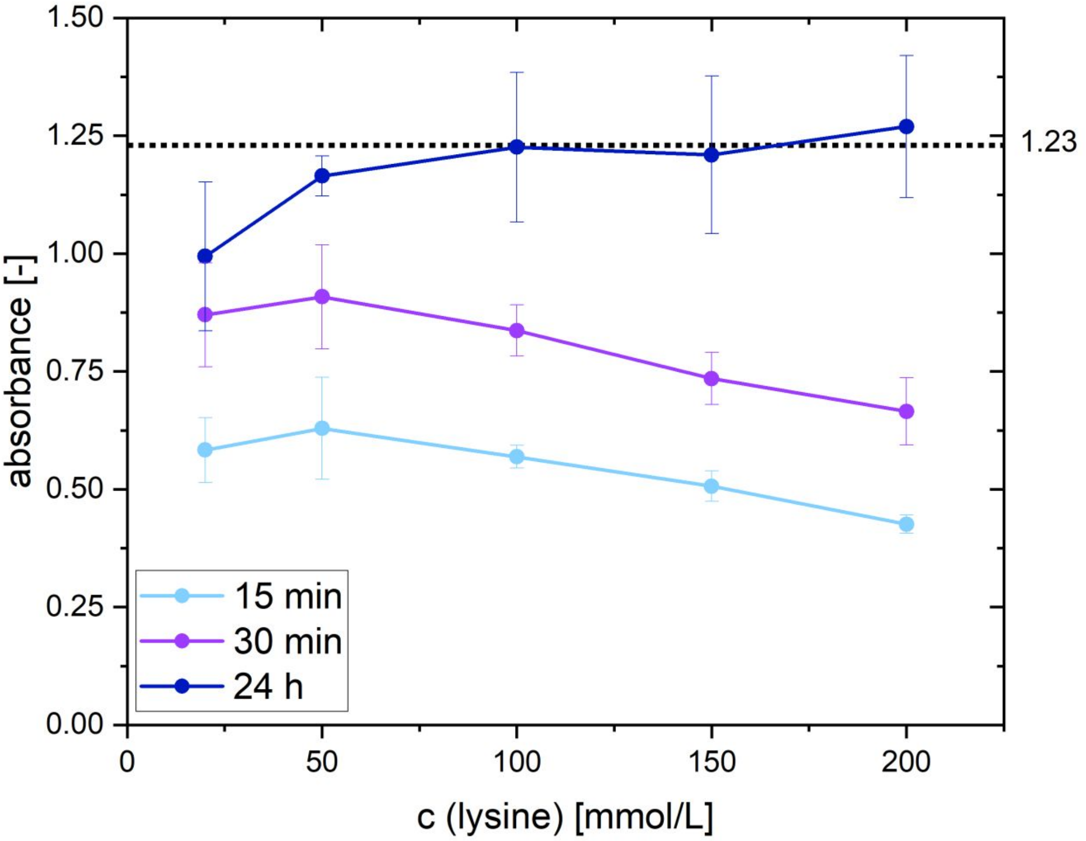

Figure S2. The absorbance of a lysine/glutaraldehyde concentration for different lysine concentrations and reaction times. For all lysine concentrations > 100 mmol/L, the absorbance after 24 h is identical (1.23), which leads to the conclusion that this absorbance is the highest possible, and occurs, when all 15 mmol/L glutaraldehyde have reacted.

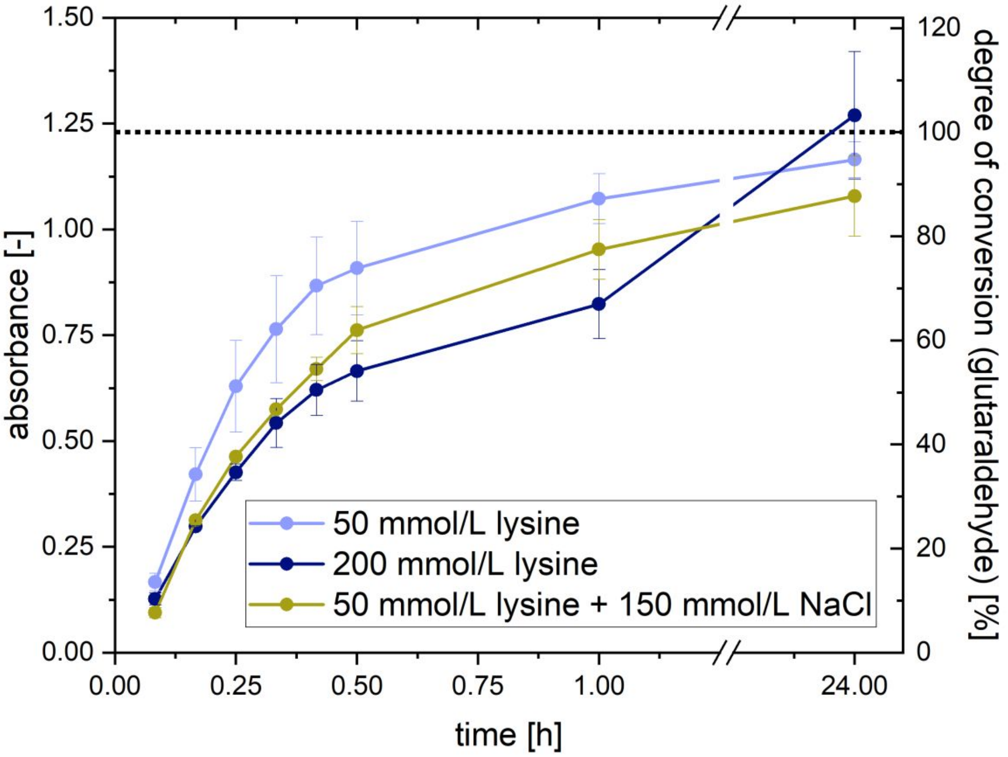

Figure S3. Time-dependent absorbance at 437 nm of 15 mmol/L glutaraldehyde with 50 mmol/L lysine (●), 200 mmol/L lysine (●), and 50 mmol/L lysine combined with 150 mmol/L NaCl (●). Reactions were performed at 25 °C.

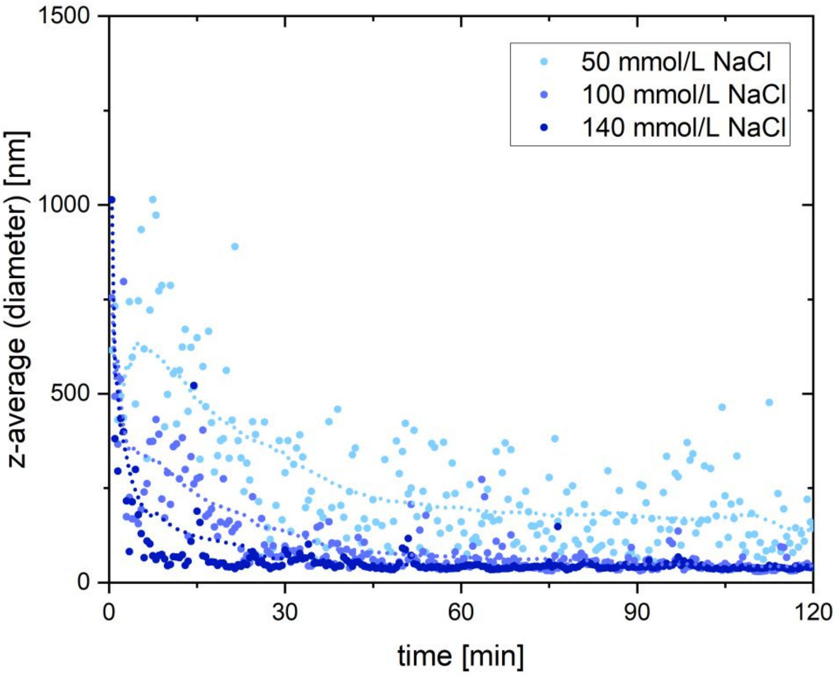

Figure S4. NaCl-induced dissolution of pseudo-fibrin studied by means of dynamic light scattering. Evolution of z-averaged particle diameter during the first 2 h after addition of NaCl is shown. The graphs were recorded at NaCl concentrations of 50 mmol/L (●), 100 mmol/L (●), and 140 mmol/L (●). Dotted lines represent an average value.

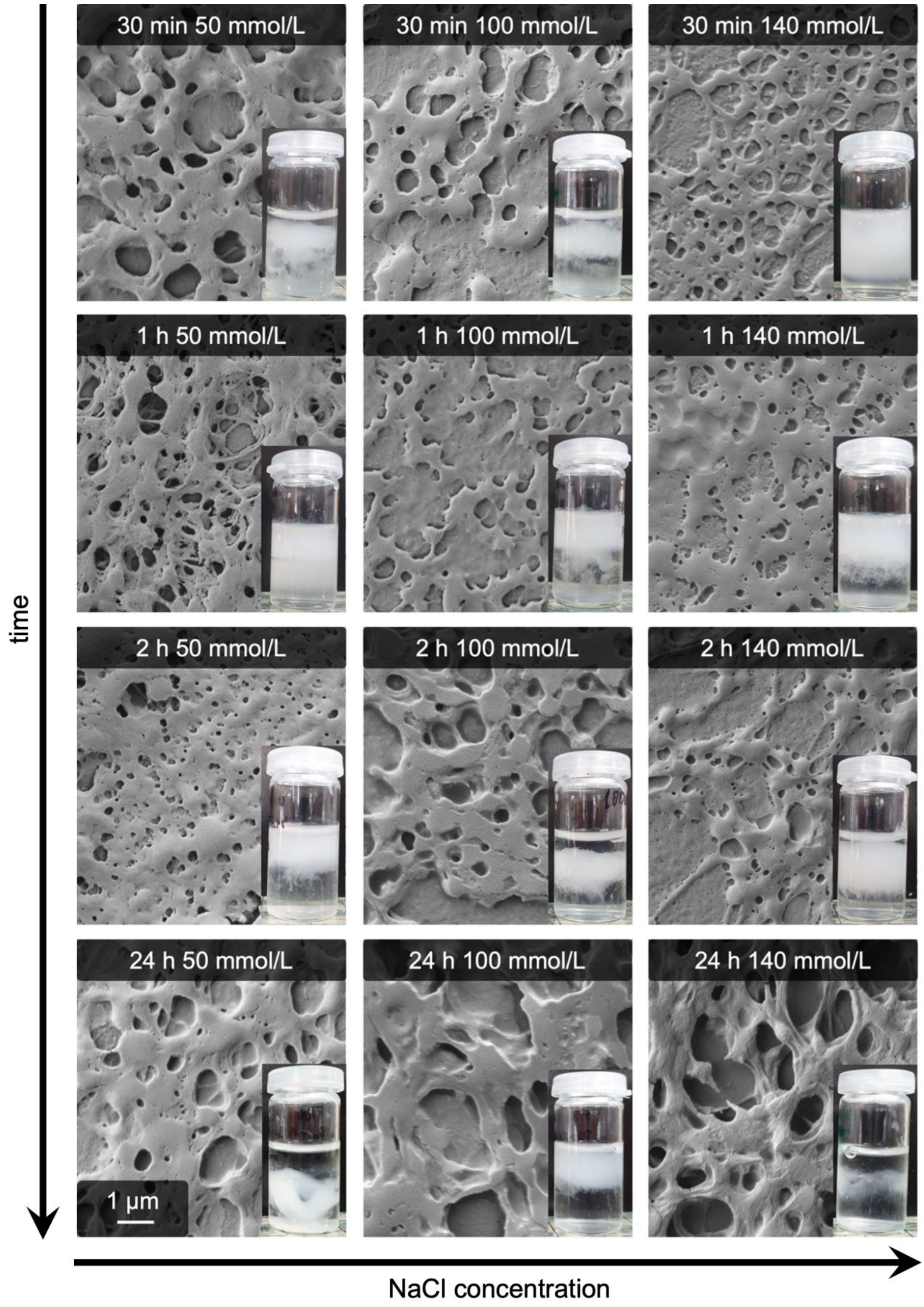

Figure S5. Effect of elevated NaCl concentrations (50 mmol/L, 100 mmol/L, 140 mmol/L) on pseudo-fibrin hydrogels and the remaining fibers. Already storage for 30 min at 50 mmol/L NaCl is sufficient to significantly damage the fibrous structure, although the gel itself remains nearly unchanged.

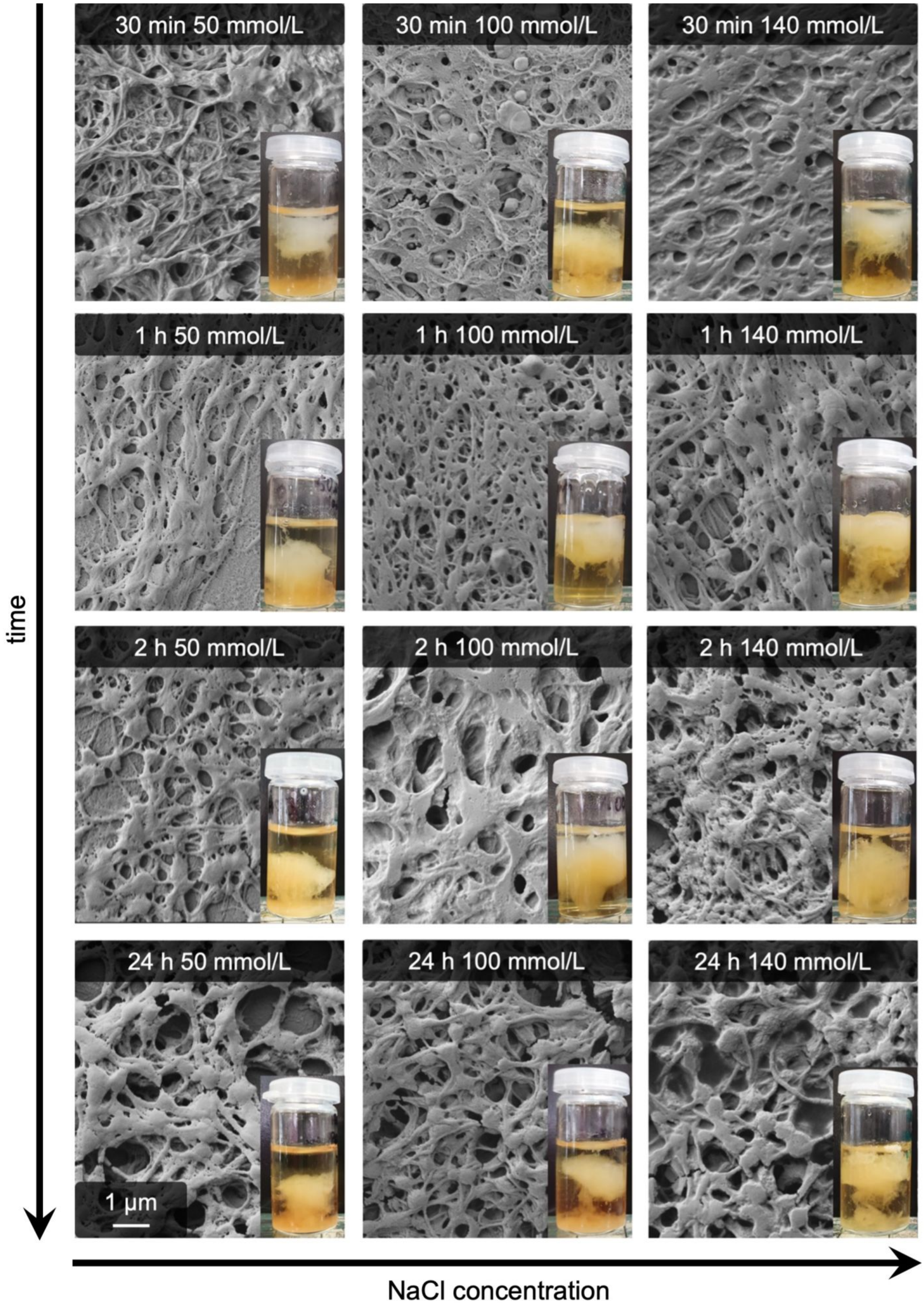

Figure S 6. Effect of elevated NaCl concentrations (50 mmol/L, 100 mmol/L, 140 mmol/L) on glutaraldehyde cross-linked pseudo-fibrin hydrogels and the remaining fibers. In all cases, the fibrous structure is retained, although especially fibers in the presence of 140 mmol/L NaCl become less defined.
